# Membrane fusion studied by colloidal probes

**DOI:** 10.1007/s00249-020-01490-5

**Published:** 2021-02-18

**Authors:** Hannes Witt, Filip Savić, Sarah Verbeek, Jörn Dietz, Gesa Tarantola, Marieelen Oelkers, Burkhard Geil, Andreas Janshoff

**Affiliations:** 1 Institute for Physical Chemistry, University of Göttingen, 37075 Göttingen, Germany; 2 Present Address: Physics of Living Systems, Vrije Universiteit Amsterdam, 1081 HV Amsterdam, The Netherlands

**Keywords:** Supported lipid bilayer, Colloidal probe microscopy, SNARE

## Abstract

Membrane-coated colloidal probes combine the benefits of solid-supported membranes with a more complex three-dimensional geometry. This combination makes them a powerful model system that enables the visualization of dynamic biological processes with high throughput and minimal reliance on fluorescent labels. Here, we want to review recent applications of colloidal probes for the study of membrane fusion. After discussing the advantages and disadvantages of some classical vesicle-based fusion assays, we introduce an assay using optical detection of fusion between membrane-coated glass microspheres in a quasi two-dimensional assembly. Then, we discuss free energy considerations of membrane fusion between supported bilayers, and show how colloidal probes can be combined with atomic force microscopy or optical tweezers to access the fusion process with even greater detail.

## Introduction

Membrane fusion plays a pivotal role in exo- and endocytosis, viral infection, and cell–cell fusion (Tamm et al. 2003; Sudhof and Rothman 2009; Chernomordik and Kozlov 2008; Cohen and Melikyan 2004; van den Bogaart et al. 2010; Smirnova et al. 2010a; Jahn et al. 2003; Hernandez et al. 2014; Rizo and Xu 2015). Opposing membranes involved in the actual fusion process are required to perform substantial shape transformations facilitated by specialized fusion proteins and supported by a tailored lipid composition (Risselada and Mayer 2020). The most well-known biological example is the $\hbox {Ca}^{2+}$- dependent fusion of synaptic vesicles with the presynaptic plasma membrane of neurons and chromaffin cells (Fasshauer et al. 1997; Stein et al. 2009; Jahn and Grubmüller 2002; Jahn et al. 2003; van den Bogaart et al. 2010). Neuronal fusion is the extremely fast—only a few microseconds—release of neurotransmitters into the synaptic cleft (Rizo and Xu 2015). Fusion in these systems is catalyzed by SNARE proteins forming a tetrameric coiled-coil complex, which lowers the energy barriers for subsequent membrane merging.

Molecular dynamics simulations and continuum models have demonstrated that membrane fusion involves a number of different intermediates and possible pathways requiring lipids and proteins to rearrange in manifold ways. In contrast, however, the information which is available from experiments is rather sparse and accessible states are often reduced to docking, hemifusion, and full fusion (Tamm et al. 2003; Cohen and Melikyan 2004; Risselada et al. 2014; Lira and Dimova 2019).

**Fig. 1:**
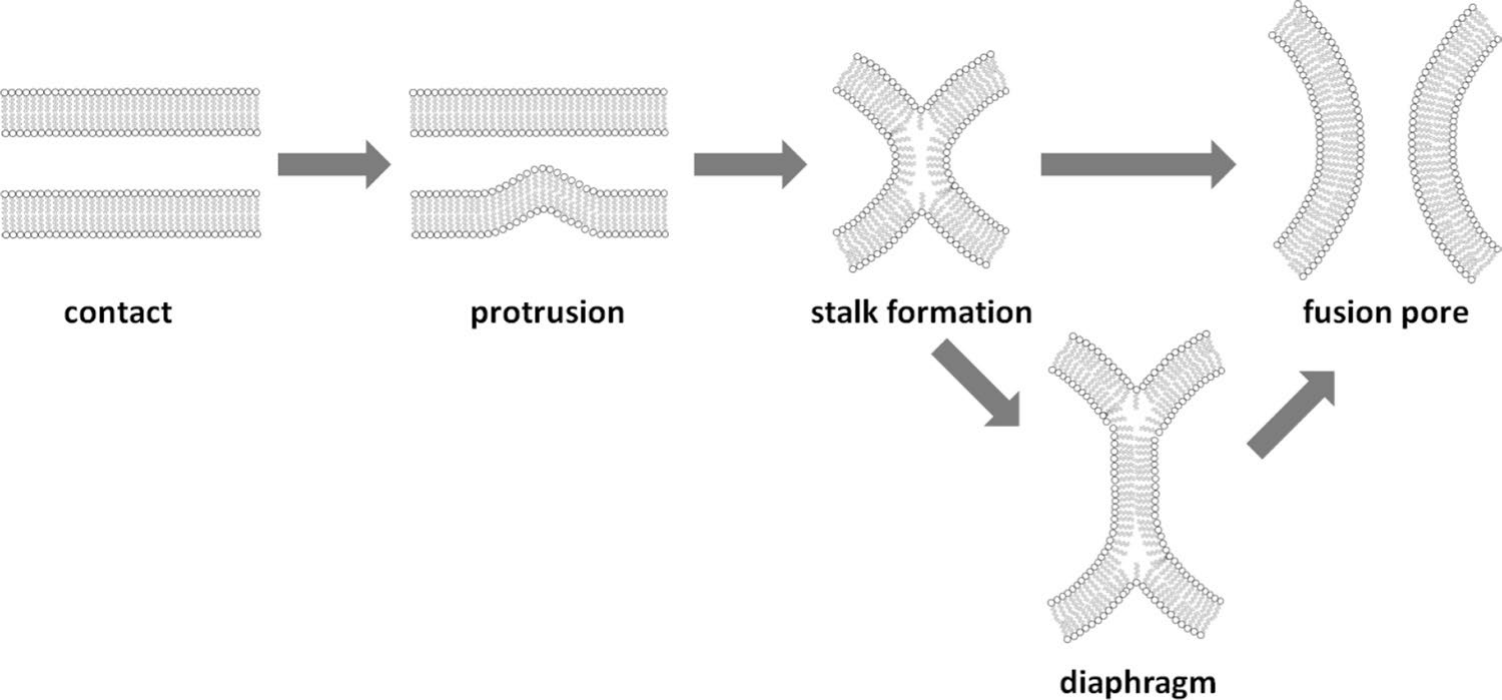
Possible intermediates during membrane fusion. Adjacent membranes form point-like protrusions in close contact. Subsequently, a hemifusion stalk forms that can either be expanded into a hemifusion diaphragm or directly create a full fusion pore (Ko et al. 2014; Chernomordik and Kozlov 2008)

Before fusion can occur, membranes must come into close contact (Fig. 1). This requires them to cross electrostatic, hydration, and steric barriers between the opposing lipid bilayers. One of these energy barriers is associated with the strong repulsive hydration force when two lipid bilayer approach each other (Leikin et al. 1987). Later, in the actual process of membrane merging, other energy-costly contributions dominate comprising initiation of stalk formation, expansion of the stalk structure, hemifusion diaphragm expansion, and, eventually, formation of the fusion pore (Jahn et al. 2003; Chernomordik and Kozlov 2008; Cohen and Melikyan 2004; Chernomordik and Kozlov 2011).

In native biological systems, a large number of pathways and regulatory proteins exist that orchestrate fusion events in an intricate way. This makes it difficult to identify the role of individual lipids and proteins (Jahn et al. 2003; Sudhof and Rothman 2009; Rizo and Xu 2015). Therefore, a number of in vitro model systems and assays have been established to identify key molecular players and the molecular composition of membranes responsible for the efficiency, speed, and location for fusion to occur.

In this review, we discuss how colloidal probes can be used to study fusion of two lipid bilayers. We start by introducing model membranes and the most commonly used vesicle-based fusion assays, before we turn to fusion assays based on membrane-coated glass microspheres. We use theoretical considerations to compare membrane fusion between vesicles to the merging of solid-supported membranes. Then, we show how colloidal probes can be combined with micromanipulation methods such as atomic force microscopy or optical tweezers to further explore the energy landscape of membrane fusion.

## Model membranes

**Fig. 2:**
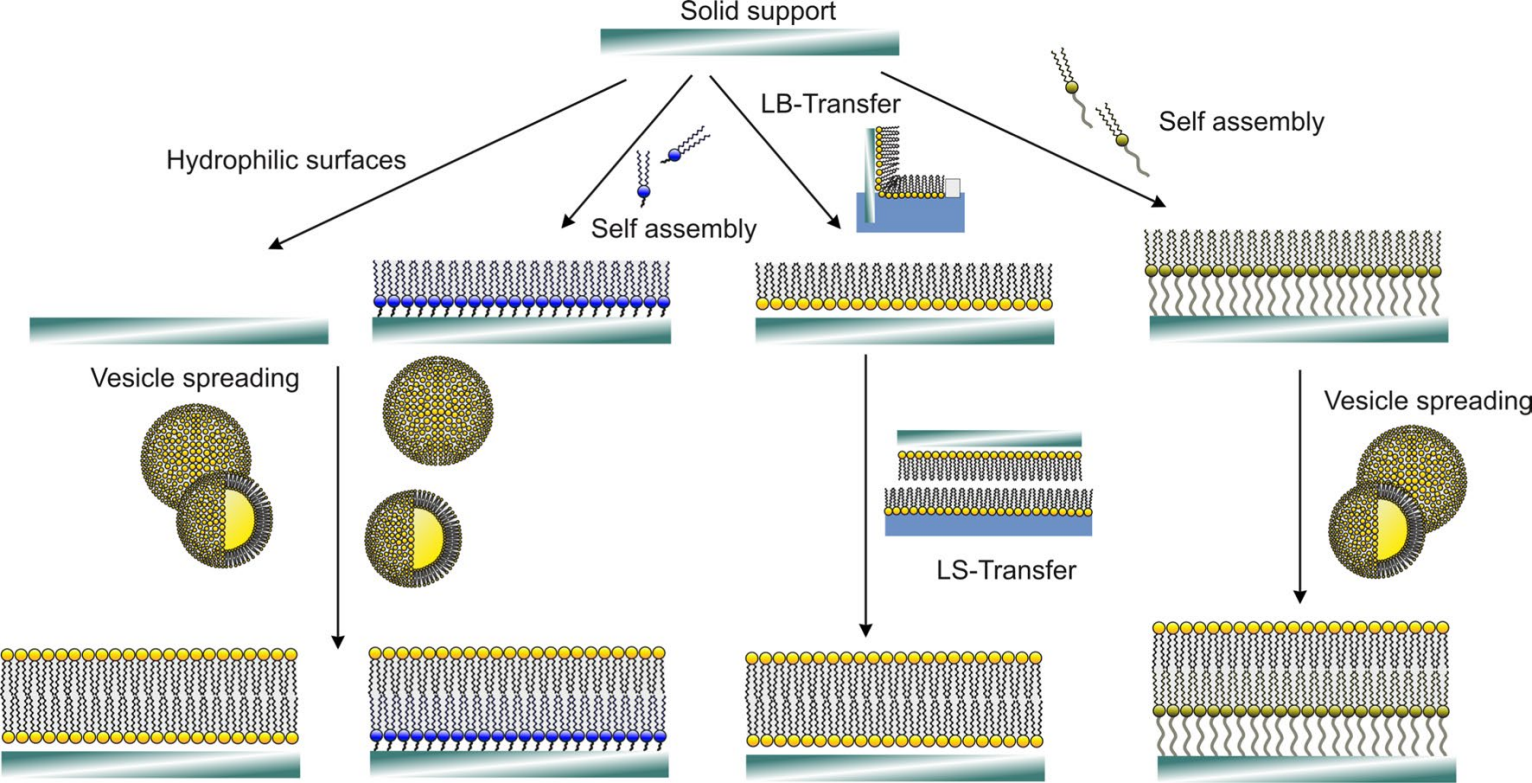
Different techniques to prepare solid-supported membranes based on chemisorption, self assembly, vesicle spreading, and Langmuir–Blodgett transfer (Janshoff and Steinem 2006)

Model membranes suitable for the study of fusion events can generally be categorized into lipid vesicles and supported lipid bilayers (Fig. 2). Liposome-based fusion assays are further subdivided depending on the size of the vesicles, i.e., small unilamellar vesicle (SUVs) with diameters below 50 nm, large unilamellar vesicles (LUVs) with sizes up to 1 $\upmu$m, and so-called giant liposomes of much larger size ranging from 10 $\upmu$m up to 100 $\upmu$m (Lira and Dimova 2019; Dimova and Marques 2019).

Supported lipid bilayers are flat, mostly planar membranes formed on a large variety of different supports including, inter alia, glass, metal (e.g., thiol-coated gold), metal oxides (most commonly silicon oxide), soft polymers, or porous supports (Steinem et al. 1996; Janshoff and Steinem 2001; Tanaka and Sackmann 2005; Richter et al. 2006; Knobloch et al. 2015; Höfer and Steinem 2010; Schwenen et al. 2015; Kuhlmann et al. 2017; Kliesch et al. 2017; Kreutzberger et al. 2019; Mühlenbrock et al. 2020). The advantage of supported lipid bilayers over liposomes is that supported bilayers can be easily combined with a multitude of analytical techniques such as ellipsometry, impedance spectroscopy, acoustic methods like the quartz crystal microbalance, scanning probe microscopy, and TIRF (total internal reflection) microscopy (Janshoff and Steinem 2006). A drawback of supported bilayers is the lack of a second aqueous compartment, which can be compensated by a polymer cushion or hybrid systems such as pore-spanning membranes (Hennesthal and Steinem 2000; Tanaka and Sackmann 2005; Kozuch et al. 2012; Frese et al. 2013).

## Vesicle-based fusion assays

**Fig. 3:**
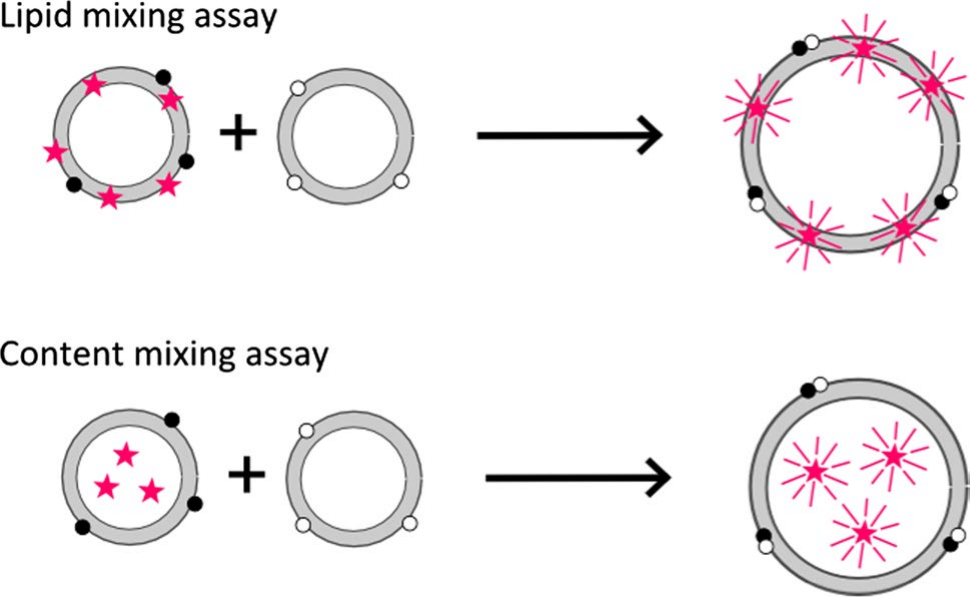
Illustration of typical lipid and content mixing assays based on dequenching. Black and white points refer to complementary fusogenic molecules. Glowing stars represent fluorescent fluorophores, while quenched fluorophores are marked as simple stars

Most fusion assays based on model membranes employ fluorescently labeled lipids or proteins as reporters of lipid or content mixing as a consequence of membrane fusion. Among the various assays, self-quenching of fluorophores in the membrane or the lumen of the liposome is most frequently used (Fig. 3). Self-quenching refers to the effect of diminished fluorescence intensity at high dye concentrations. Therefore, dilution of dyes in the course of fusion results in an increase in fluorescence. This might be indicative of membrane merging of one or two leaflets, but might also indicate release of the vesicle content if the fluorophores are water soluble and enclosed in the vesicle lumen. Typically, the fraction of lipid dyes in the membrane to achieve self-quenching can be up to 5 mol% of the membrane lipids, whereas the concentration of encapsulated water-soluble dyes has to be higher than 50 mM (Garcia 1992; Nieva et al. 1994; Lira and Dimova 2019). Since these assays heavily rely on the fluorescence readout, they may be compromised by effects such as spontaneous transfer of the fluorescent probe to unlabeled membranes, interactions between fluorophores with the proteins of interest, or quenching of fluorescence by adjacent proteins in the membrane.

Alternatively, fusion assays rely on externally added fluorescence quenchers. This usually requires lower dye concentrations, which is beneficial in avoiding possible artefacts. Fusion will then result in an increase or decrease of fluorescence depending on the location of the quencher (Kreye et al. 2008; McIntyre and Sleight 1991). Since lipid bilayers are impermeable to polar molecules typical quenchers like dithionite can be used to selectively quench the outer leaflet of LUVs. Quenching is in most cases irreversible and can therefore be used to distinguish different states. If two separate populations of LUVs are used—one unlabeled and the other one labeled at self-quenching concentrations—the labeled LUVs can be irreversibly quenched at the outside. Later, the quencher is removed by size-exclusion chromatography and the two populations of LUVs are mixed. An increase of fluorescence after the addition of the fusogen to two populations of vesicles indicates full fusion between the vesicles, since the fluorescence on the inner leaflet is no longer self-quenched. If, however, an increase in fluorescence is detected in the absence of chemical quenchers, but vanishes when a quencher is added, one might conclude that only hemifusion (mixing of the outer leaflet) took place (Lira and Dimova 2019).

Along similar lines, pyrene–excimer formation can be used to detect fusion events quantitatively (Stegmann et al. 1993). Notably, all these techniques have only a small linear window, where fluorescence intensity is proportional to fluorophore concentration limiting their use to provide reliable data over a large concentration range.

Another popular and very sensitive way to detect fusion and possible intermediate states relies on fluorescence resonance energy transfer (FRET), where the external quencher is replaced by another fluorophore acting as an FRET acceptor (Struck et al. 1981). After successful fusion, donor or acceptor fluorescence can increase or decrease depending on the locations of the fluorophores. This method allows for the determination of kinetic information and, therefore, gives access to the actual fusion mechanism in a time-resolved manner. Some of the typical problems with FRET-based methods comprise wrong stoichiometry, insufficient protein labeling, or over-loading of the acceptor with fluorophores leading to interaction of the donor with several acceptors (Becker 2012).

Detection of docking events in vesicle-based fusion assays is cumbersome and might require additional microscopy techniques. Since vesicle-based fusion assays are usually performed as bulk experiments in a fluorimeter aggregation of vesicles often leads to light scattering rendering quantitative measurements a difficult task. On the bright side, however, dynamic light scattering can be used as a first indicator of interaction and fusion of vesicles in the absence of fluorescent labels (Castorph et al. 2011; Yang et al. 2015).

The concepts above can generally be used for any membrane topology such as vesicles of any size, solid-supported membranes, or even living cells. They might be used in ensemble measurements employing fluorescence spectrometers as well as single vesicle arrangements using microscopy techniques.

## Fusogens

**Fig. 4:**
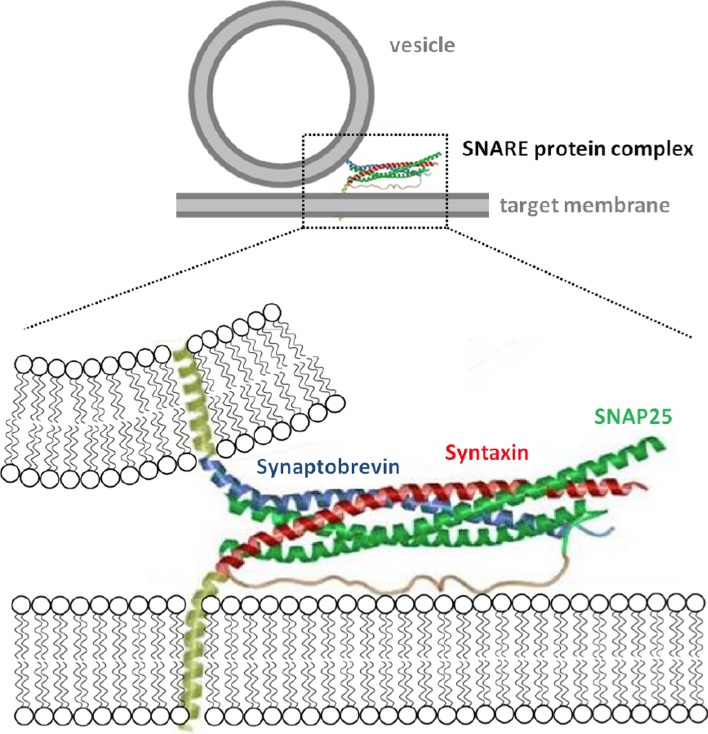
Schematic illustration of the SNARE complex mediating membrane fusion. The core SNARE complex consists of syntaxin (red), synaptobrevin (blue), and SNAP25 (green) anchored in lipid bilayers with transmembrane domains (yellow) (adapted from Sutton et al. (1998))

Fusogens facilitate membrane merging by providing the energy to overcome the energy barriers of membrane fusion (Marsden et al. 2011; Podbilewicz 2014; Hernández and Podbilewicz 2017). Fusion of two membranes requires close proximity (Jahn et al. 2003; Sudhof and Rothman 2009; Risselada and Mayer 2020). The hydration barrier, i.e., the desolvation of the lipid bilayers, is one of the major reasons fusion of lipid bilayers is rarely observed in the absence of fusogens (Smirnova et al. 2010b). Later, in the process, more energy barriers associated with stalk formation and fusion pore widening occur depending on the pathway and molecular composition (Risselada et al. 2014; Risselada and Mayer 2020). The rates at which these processes occur in vitro are usually extremely low and typically require the action of specialized proteins or peptides to lower activation barriers and thereby to change the free energy landscape in a defined way. This ensures that fusion can occur on reasonable time scales. Generally, any molecule or ion triggering fusion of lipid bilayers is called a fusogen.

In native cellular systems, fusion is usually mediated by a complementary set of proteins located in opposing membranes, while viruses like the influenza virus or human immunodeficiency virus (HIV) display all necessary proteins in their own membrane (Eckert and Kim 2001; Hernández and Podbilewicz 2017). In cells, the most frequently occurring fusogens belong to a protein family called SNAREs (soluble *N*-ethylmaleimide-sensitive fusion attachment protein receptors) (Jahn and Scheller 2006). The process involves SNARE proteins to assemble into a ternary parallel-oriented coiled-coil bundle consisting of syntaxin and two SNAP25s on one membrane and synaptobrevin on the opposing bilayer (Jahn and Scheller 2006). Zippering of an eight-heptad repeat segment into a superhelix has been shown to be responsible for the highly stable coiled-coil motif (Fig. 4) (Pobbati et al. 2006).

**Fig. 5:**
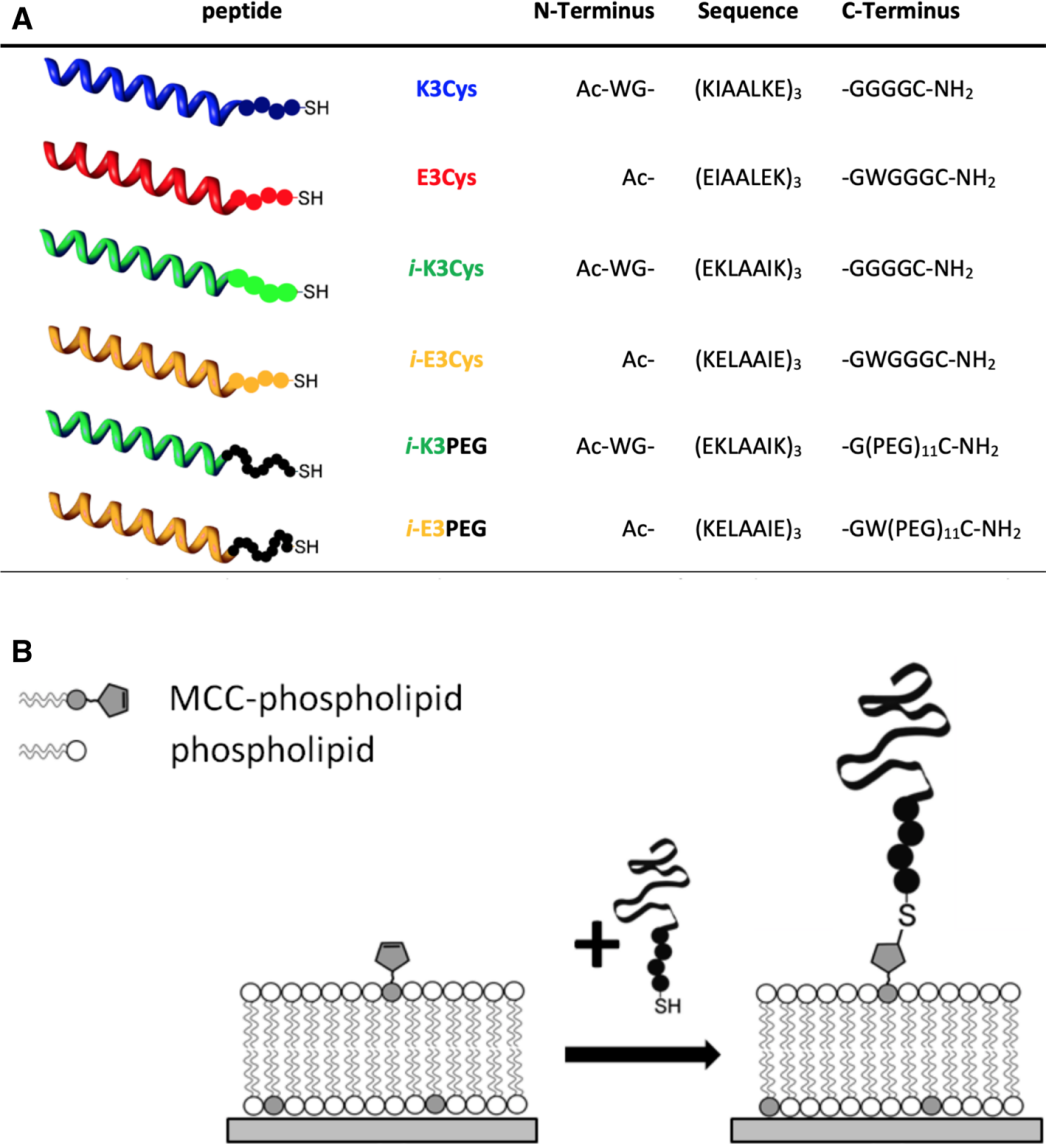
**a** SNARE mimics based on coiled-coil forming peptides with acetylated N-terminus and amidated C-terminus. **b** Illustration of the envisioned in situ coupling reaction between MCC-phospholipid (MCCDOPE: 1,2-dioleoyl-*sn*-glycero-3-phosphoethanolamine-N-[4-(*p*-maleimidomethyl)cyclohexane-carboxamide])) on an SSM (solid-supported membrane) with a cysteine-terminated peptide (adapted from Pähler et al. (2012))

It turns out that there exist simpler solutions to fulfil the prerequisite to act as a fusogen. Due to their ability to lower the hydration barrier as the initial step in fusion, polyvalent ions, charged peptides, artificial polymers, or nucleic acids with strong membrane affinity might serve as possible fusion initiators, as well (Stengel et al. 2007; Marsden et al. 2009, 2011; Lygina et al. 2011; Pähler et al. 2012; Flavier and Boxer 2017). However, as most membrane-active peptides and poly-electrolytes, these fusogens might also induce unwanted membrane rupture instead of ‘clean’ fusion.

A very popular artificial system uses the so-called coiled-coil peptides mimicking native viral proteins or SNAREs (Marsden et al. 2009, 2011; Lygina et al. 2011; Meyenberg et al. 2011; Pähler et al. 2012). Coiled-coil superhelices are formed by two or more $\upalpha$-helices wrapping around each other (Grigoryan and Keating 2008). It is a common structure in eukaryotic cells found also in different contexts such as muscle proteins and intermediate filaments such as keratins. Especially, in membrane fusion, coiled-coil interactions are widespread (Rose and Meier 2004). For instance, enveloped viruses such as HIV and influenza rely on proteins that display coiled-coil sequences as the major folding motif (Eckert and Kim 2001).

**Fig. 6:**
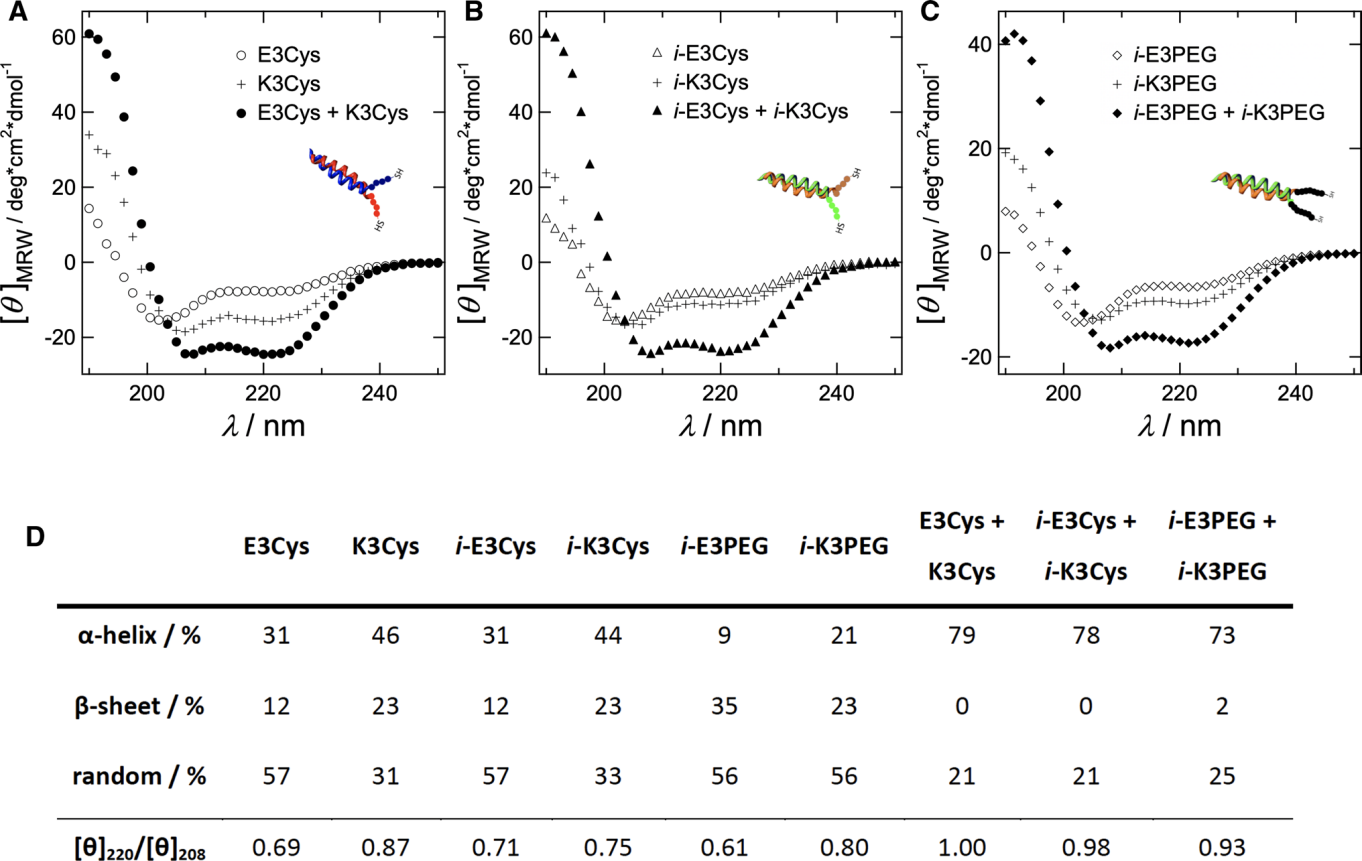
CD spectra of individual peptides and corresponding heterodimers. **a** E3Cys (open circles), K3Cys (crosses), and their 1:1 mixture (filled circles). **b** *i*-E3Cys (open triangles), *i*-K3Cys (crosses) and their 1:1 mixture (filled triangles). **c** *i*-E3PEG (open squares), *i*-K3PEG (crosses), and their 1:1 mixture (filled squares). **d** Fractions of major secondary structures before and after formation of coiled-coil dimers (adapted from Pähler et al. (2012))

Based on this iconic conformation, a broad variety of minimal fusion systems have been created to mimic membrane fusion, for instance, based on DNA (deoxyribonucleic acid), PNA (peptide nucleic acid), or short helical peptides that can form dimeric coiled-coil complexes (Stengel et al. 2007; Marsden et al. 2011; Lygina et al. 2011; Meyenberg et al. 2011; Pähler et al. 2012). All these model systems share the feature that dimerization follows a zipper-like mechanism comparable to SNARE proteins, however, often forming merely dimers and not tetramers. While peptides are designed to form short heterodimeric coiled-coil helices (called E- and K-peptide), single-stranded DNA and PNA sequences hybridize to form double helices (Flavier and Boxer 2017; Stengel et al. 2007; Hubrich et al. 2018). The role of anchoring of these recognition units in the lipid bilayer and the associated force transmission remains an unsolved question. Meyenberg et al. (2011) used a transmembrane protein linker derived from native SNARE proteins to anchor the recognition peptide sequence into the bilayer, while Marsden et al. (2009) relied on a phospholipids as bilayer anchors equipped with a short PEG spacer to provide some flexibility that should enhance the probability to form dimers. Both groups were able to show significant lipid mixing as well as some content mixing, indicating full membrane fusion. Along these lines, McNew et al. (2000) showed that the type of anchor is important for high fusion efficiency, with transmembrane linkage being more effective than lipid anchoring. Besides, the orientation of the recognition sequences influences fusion efficiency, as well, as shown by Lygina et al. (2011). Higher fusion efficiency was reported for PNA dimers exhibiting parallel orientation. Similarly, only parallel DNA superhelices led to a significant number of fusion events, while for the anti-parallel heterodimerization, docking prevailed (Kumar et al. 2015).

**Fig. 7:**
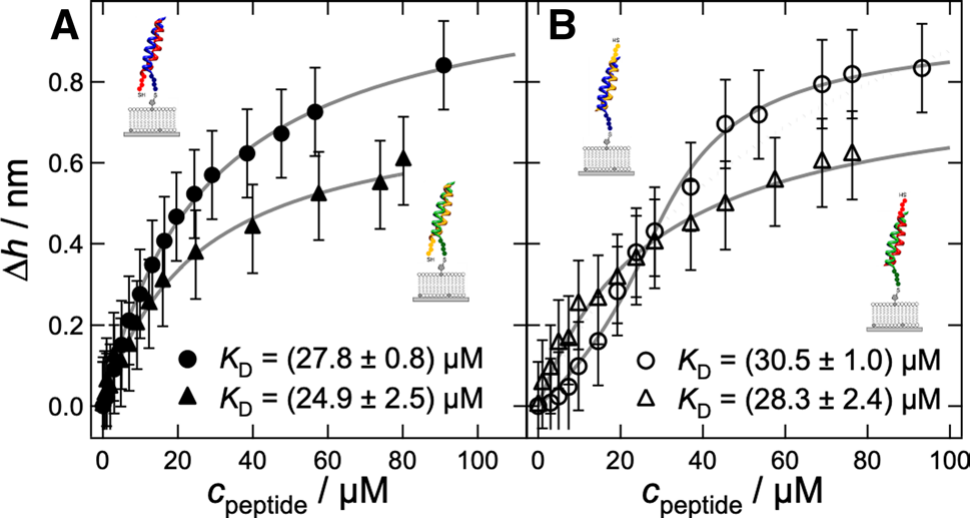
Adsorption isotherms of E-peptides recognized by surface bound K-lipopeptides (K-LP) obtained from ellipsometry measurements. Bilayers consisted of DOPC/MCCDOPE 97:3, and K-peptides were covalently coupled to the bilayer surface. **a** Parallel coiled-coil formation. Solid circles: LP-K3Cys + E3Cys; solid triangles: LP-*i*-K3Cys + *i*-E3Cys; data were fitted according to a Langmuir isotherm. **b** Antiparallel coiled-coil formation. Open circles: LP-K3Cys + *i*-E3Cys; open triangles: LP-*i*-K3Cys + E3Cys. A Langmuir isotherm was fit to the data for LP-*i*-K3Cys + E3Cys, while a Bragg–Williams isotherm was fit to data for LP-K3Cys + *i*-E3Cys (fits:gray line) (adapted from Pähler et al. (2012))

To facilitate membrane fusion, the interaction energy of these artificial fusogens needs to be high enough to overcome the energy barriers towards fusion. Therefore, it is necessary to thoroughly characterize their propensity to dimerize, both in solution as well as in the context of lipid membranes. Figure 5 shows typical sequences used to study docking and fusion in peptide-based model systems (Marsden et al. 2009; Pähler et al. 2012, 2013; Bao et al. 2013). The formation of coiled-coil dimers oligomers from E- and K-peptides in solution can be easily monitored by means of CD spectroscopy or IR spectroscopy (Pähler et al. 2012). Figure 6 shows CD spectra of the following mixtures: K3Cys and E3Cys, *i*-K3Cys and *i*-E3Cys as well as *i*-K3PEG with *i*-E3PEG (see also Fig. 5). Each set of peptides showed a clear propensity to form $\upalpha$-helices upon dimerization. The free energy of binding between E- and K-peptides can be measured by a number of different methods such as ATR-IR, CD spectroscopy, SPR, and ellipsometry (Pähler et al. 2012). Figure 7 shows binding isotherms obtained by ellipsometry, essentially measuring the change in layer thickness or coverage upon binding of soluble peptide to lipopeptides. Regardless of the peptides’ sequences, all isotherms displayed similar $K_\mathrm {D}$-values ranging between 25 and 31 $\upmu$M, substantially smaller than those reported for SNARE zippering Gao et al. (2012). This is mainly due to the reduced length. Compared with peptide dimerization in solution, the $K_\mathrm {D}$ values of coiled-coil complexes at the membrane surface were larger by approximately one order of magnitude. The corresponding loss in free-energy of association can be attributed to a loss of translational entropy at the surface in contrast to dimerization in solution (Ben-Tal et al. 2000).

## Colloidal systems: bead-based fusion assay

**Fig. 8:**
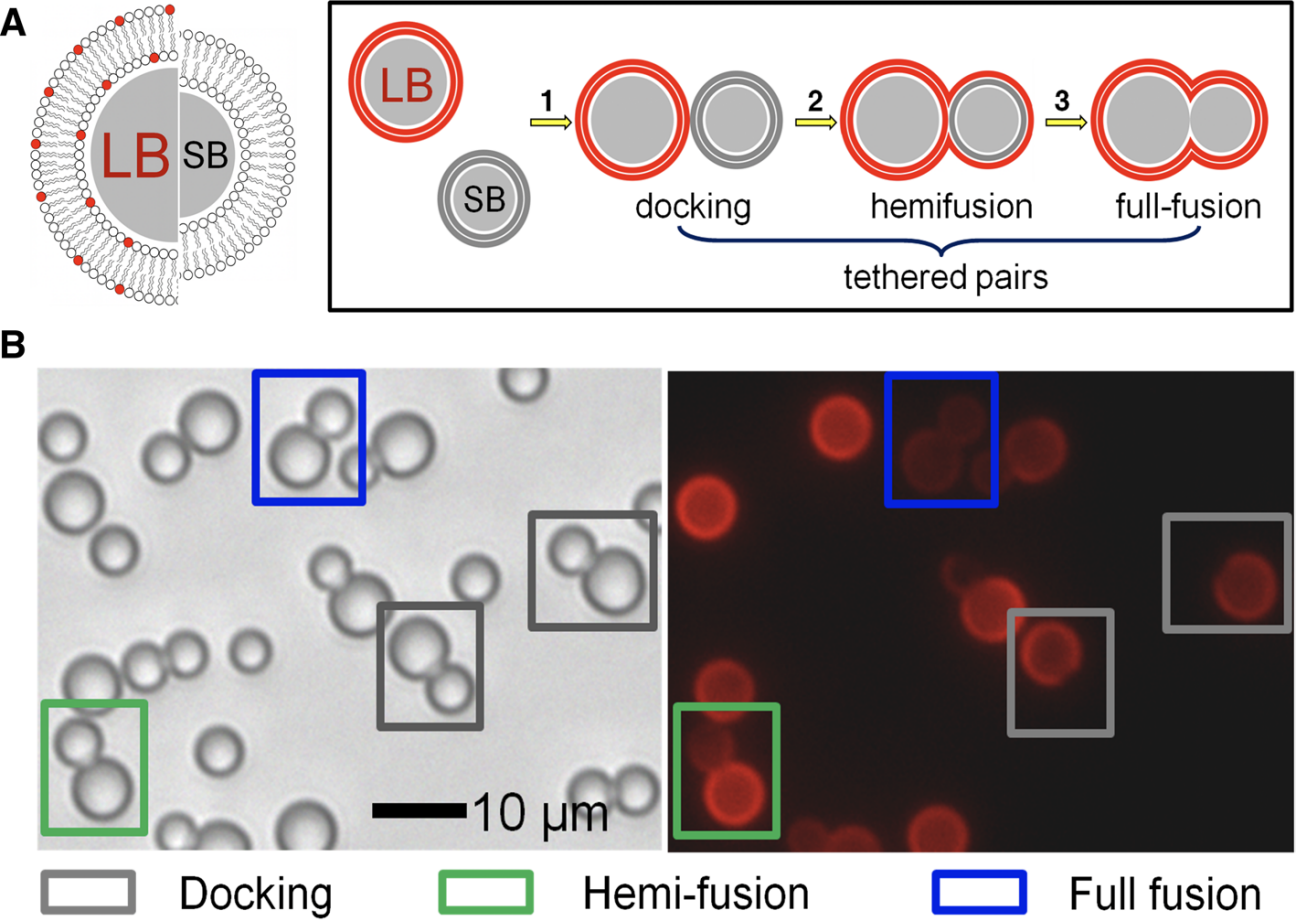
**a** Illustration of large and small silica beads coated with a lipid bilayer (LB, diameter 6.5 $\upmu$m and SB, diameter 4.7 $\upmu$m). Only the large bead (LB) is coated with a fluorescently labeled membrane. The distinct intermediate states such as docking (1), hemifusion (2), and full fusion (2) can be distinguished unequivocally by a combination of bright-field and fluorescence microscopy. **b** Two micrographs showing a bright-field (left) image and from the same spot also a fluorescence image (right) of LB-*i*-K3 and SB-*i*-E3 on a glassy surface. Docked pairs (gray box), hemifused pairs (green box), as well as fully fused pairs (blue box) are discernible (from Bao et al. (2013))

An alternative fusion assay solving some of the drawbacks of vesicle-based assays discussed in Sect. 3 is based on membrane-coated spheres in a 2D assembly allowing for the observation of docking, hemifusion, and full membrane merging of both leaflets (Bao et al. 2013; Savić et al. 2016). This robust and versatile concept, which works with a minimum of fluorophores, is illustrated in Fig. 8.

**Fig. 9:**
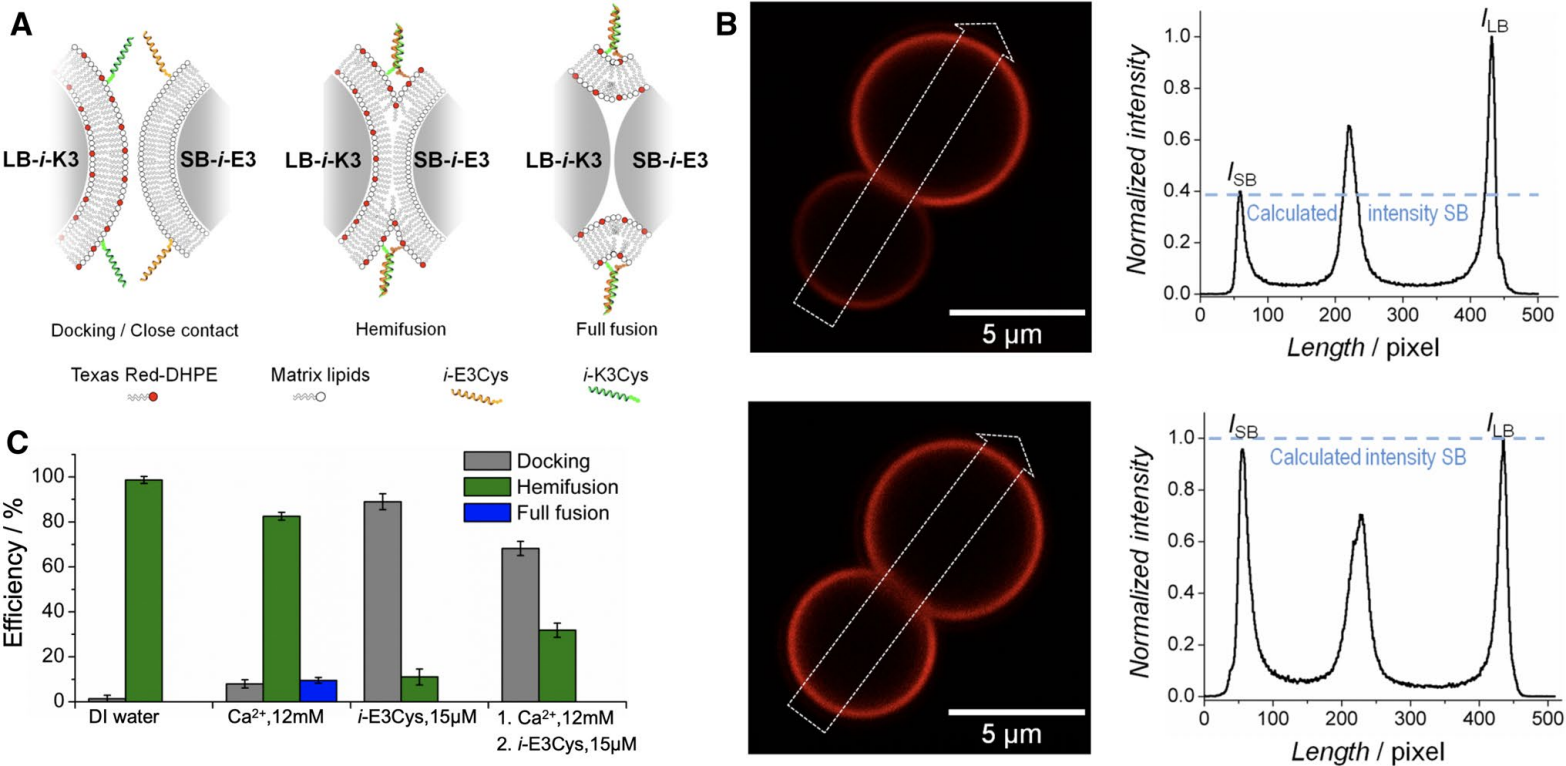
**a** Scheme of the discernible steps of the bead assay comprising approach/docking (left), hemifusion(center), and full fusion (right) of membrane-coated beads (LB-*i*-K3/SB-*i*-E3) triggered by creation of parallel coiled-coil peptide dimers. **b** Fluorescence intensity integrated over a rectangular profile (white arrow) across a bead-pair (LB-*i*-K3/SB-*i*-E3). The dashed line correspond to calculated intensities $I_\mathrm {SB}$. Hemifusion (top) is identified by an intensity ratio between LB:SB of 1:0.4. Full fusion of both leaflets (bottom) requires an equal distribution of fluorophores (LB:SB 1:0.95). **c** Occurrence of various events comprising docking (gray), hemifusion (green), and full fusion (blue) of LB-*i*-K3/SB-*i*-E3 pairs depending on the presence of $\hbox {Ca}^{2+}$-ions and administration of an soluble inhibitor (*i*-E3Cys) (from Bao et al. (2013))

The general strategy relies on the concept that two populations of monodisperse silica microspheres differing in size and membrane composition can be distinguished merely by size. The membranes are deposited on the beads’ surfaces by vesicle spreading of SUVs or LUVs (Baksh et al. 2004; Steinem and Janshoff 2004; Bao et al. 2013). Heterogeneous bead-pairs consist of one LB (large bead, diameter 6.5 $\upmu$m, labeled) and one SB (small bead, diameter 4.7 $\upmu$m, not labeled) in close contact. These pairs form due to the Brownian motion of the beads on the surface. The three most prominent steps during membrane fusion, i.e., docking, hemifusion, and full fusion, were identified unequivocally by monitoring both the position of the beads and the fluorescence intensity of the lipid membranes. For instance, a fluorescent membrane surrounding a large bead in contact with a dark small bead (only visible in bright-field microscopy) is identified as a pure docking event, while an SB with half of the intensity found on the LB is indicative of merging of the outer leaflet, i.e., hemifusion. Full fusion corresponds to both beads displaying the same fluorescence intensity (Fig. 9). Efficiencies of docking, hemifusion, and full fusion are shown in Fig. 9c. It was found that full fusion rarely occurred, while in the absence of calcium ions, hemifusion prevailed to 99%. Inhibitors that form dimers with the surface bound K-peptides abolished full fusion entirely and the system remained mainly docked.

### Membrane-coated beads: energetics

**Fig. 10:**
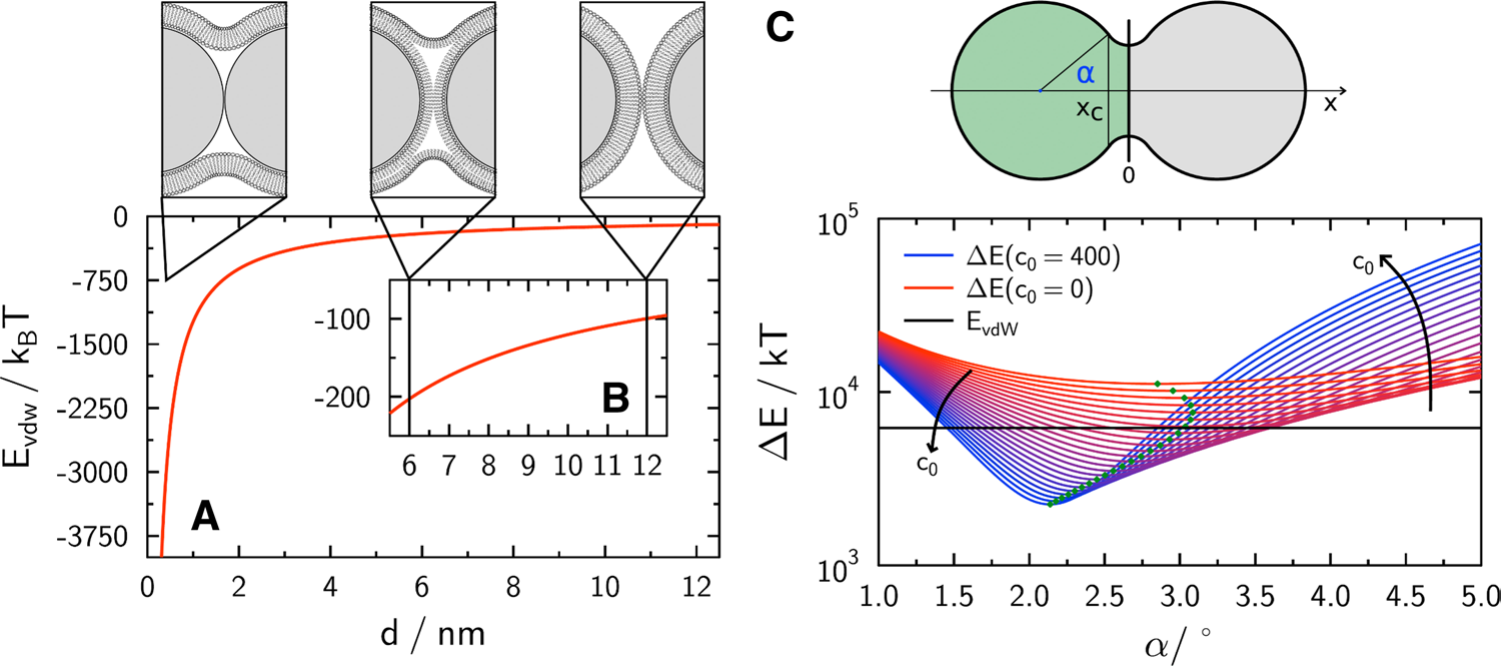
**a** Interaction energy (van der Waals) between two silica beads as a function of distance between the spheres. **b** Zoom-in figure corresponding to the different intermediate states sketched above. **c** Top: definition of contact angle $\alpha$. $\alpha$ denotes the angle between the *x* axis, which connects the center of the two spheres and the point where delamination of the membrane from the sphere begins. Bottom: overall change in energy due to merging of the membranes on two membrane-coated silica spheres assuming that a torus has been formed by the merged bilayer in the contact zone. The spontaneous curvature $c_0$ is color-coded. The black line represents the van der Waals energy released after direct contact of the two spheres. If the total energy change lies below this line, fusion is exergonic. Green dots indicate the energy minimum for a given spontaneous curvature $\left[ \upmu \mathrm {m}^{-1}\right]$, $\kappa = 20\,k_\mathrm {B}T$ and ${\bar{\kappa }} = 0$ (from Savić et al. (2016))

In vesicle fusion, the driving force is essentially the release of bending energy, which is independent of the vesicle’s radius and has typically values on the order of 500 $k_\mathrm {B}T$. In contrast, the driving force for fusion in a bead experiment, where the two opposing membrane merge without removal of a curved structure, originates from attractive van der Waals interactions of the two identical silica beads in close contact:

1 $$\begin{aligned} E_{\mathrm {vdW}}(d)= &\, \frac{-A_\mathrm{H}}{6}\left\{ \frac{2 R_\mathrm{S}^{2}}{\left( 4 R_\mathrm{S}+d\right) d}+\frac{2 R_\mathrm{S}^{2}}{\left( 2 R_\mathrm{S}+d\right) ^{2}}\right. \nonumber \\&\left. +\ln \frac{\left( 4 R_\mathrm{S}+d\right) d}{\left( 2 R_\mathrm{S}+d\right) ^{2}}\right\} , \end{aligned}$$

where *d* is the distance between the two beads, $R_\mathrm {s}$ their radius, and $A_\mathrm {H}$ the Hamaker constant of silica. During formation of the fusion pore, the membrane delaminates from the surface at the contact zone and thereby changes its topology permanently. Delamination from the silica surface requires to raise an equivalent amount of energy to reverse adhesion of the bilayer to the substrate. Assuming the same geometry, as shown in Fig. 10, one arrives at a delamination energy $E_{\mathrm {del}}$ of:

2 $$\begin{aligned} E_{\mathrm {del}}=4 \pi \gamma R_\mathrm{S}^{2}(1-\cos \alpha ), \end{aligned}$$

with $\gamma$ being the surface energy (typically around 0.1 mN m^-1^) and $\alpha$ being the contact angle (Savić 2015; Savić et al. 2016). Surface energies can be measured by pulling membrane tethers from a solid-supported membrane and relating tether forces to the membrane tension, which originates predominately from the adhesion energy per unit area (Kocun and Janshoff 2012). The shape of the membrane after fusion has to be considered as well to estimate the overall energy change of the membrane $E_{\mathrm {mem}}$ comprising membrane bending and area dilatation:

3 $$\begin{aligned} E_{\mathrm {mem}}=\int _{M} \mathrm {d} A\left\{ \frac{1}{2} \kappa \left( 2 H-c_{0}\right) ^{2}+{\bar{\kappa }} K +\gamma \right\} , \end{aligned}$$

where *H* denotes the mean curvature and *K* is the Gaussian curvature (Savić 2015; Savić et al. 2016). Assuming a toroidal shape of the membrane around the contact zone, the contribution originating from bending is approximated by:

4 $$\begin{aligned} \Delta E_{\text{ bend } }\approx & \, 2 \pi \kappa \cos \alpha \left[ 4-\left( c_{0} R_\mathrm{B}-2\right) ^{2}\right] \nonumber \\&- \pi \kappa \frac{R_{T}}{R_\mathrm{B}}(2 \alpha -\pi )\left( c_{0} R_\mathrm{B}-1\right) ^{2} \nonumber \\&- 8 \pi \kappa -4 \pi {\bar{\kappa }}, \end{aligned}$$

where $c_0$ is the spontaneous curvature of the membrane, $\kappa$ the bending modulus, and ${\bar{\kappa }}$ the saddle splay modulus. The approximation holds if the major torus radius $R_\mathrm {T}$ is larger than the minor torus radius $R_\mathrm {B}$ and the contact angle $\alpha$ is small. Since the contact region is very small compared to the overall surface area of the two beads, area dilation is negligible and $E_\text {deform}\approx \Delta E _\text {bend}$. A toroidal approximation has previously been used for capillary bridges between spheres with small liquid–solid contact angles (Lian and Seville 2016).

Figure 10c shows the change in energy $\Delta E \approx E_{\text {vdw}}+E_{\text {del}}+\Delta E_{\text {bend}}$ upon full fusion of membrane-coated silica spheres as a function of $\alpha$ assuming the formation of a torus between the two spheres (Savić et al. 2016). The competition between bending energy and delamination leads to a free energy minimum depending on the spontaneous curvature. Importantly, if membranes on the surface do not display a net spontaneous curvature, the gain through changes of the van der Waals interaction could be insufficient to generate enough energy to ensure fusion. However, if the lipids or proteins of the membrane display negative spontaneous curvature on the order of 100 $\upmu$m^-1^, the fusion process becomes energetically favorable. In conclusion, it is important to ensure a lipid membrane composition that bears considerable spontaneous curvatures to foster fusion in this type of experiment. Note that phosphatidylethanolamine and cholesterol are frequently used in lipid compositions with high fusion yield providing the required spontaneous curvature. It is also conceivable that initially symmetrical lipid bilayers without net spontaneous curvature can lead to full fusion by flip-flop of lipids or lateral phase separation prior to the formation of the fusion pore.

### Colloidal probe microscopy

By combining colloidal probes with micromanipulation techniques like optical tweezers or atomic force microscopy (AFM), they become an even more powerful tool to study membrane fusion. The main advantage is that these methods provide experimental data on the energy landscape and the involved mechanical forces.

Pioneering work comes from Israelachvili and coworkers who first demonstrated membrane fusion of supported lipid bilayers using a surface force apparatus (Helm et al. 1992; Israelachvili et al. 2010; Lee et al. 2015). Abdulreda et al. (2009) used a colloidal force microscope to examine the interaction forces between reconstituted SNARE derivatives by varying the loading rate. The authors assigned mechanical instabilities in the approach curve of two membrane-coated surfaces to the fusion of lipid bilayers. A different approach has been realized by Brouwer et al. (2015) and Keidel et al. (2016), who used optical tweezers to manipulate membrane-coated beads to study some aspects of membrane fusion. Keidel et al. showed that the progression of fusion is mirrored in the three-dimensional position histogram of a bead in an optical trap and in its rate of diffusion. The authors observed the following fusion intermediates: transient fusion, formation of a stalk, hemifusion, and completion of a fusion pore. Fusion intermediates displayed axially but not laterally confined bead motion. However, lateral diffusion was slowed down due to presence of a stalk-like connection between the two membranes. More recently, Sorkin et al. (2019) used the same approach to compare synaptotagmin and Doc2b induced membrane remodeling. They found that the soluble C2AB domain of synaptotagmin1 changes both the probability and also the strength of membrane–membrane interactions as a function of calcium content and protein concentration.

**Fig. 11:**
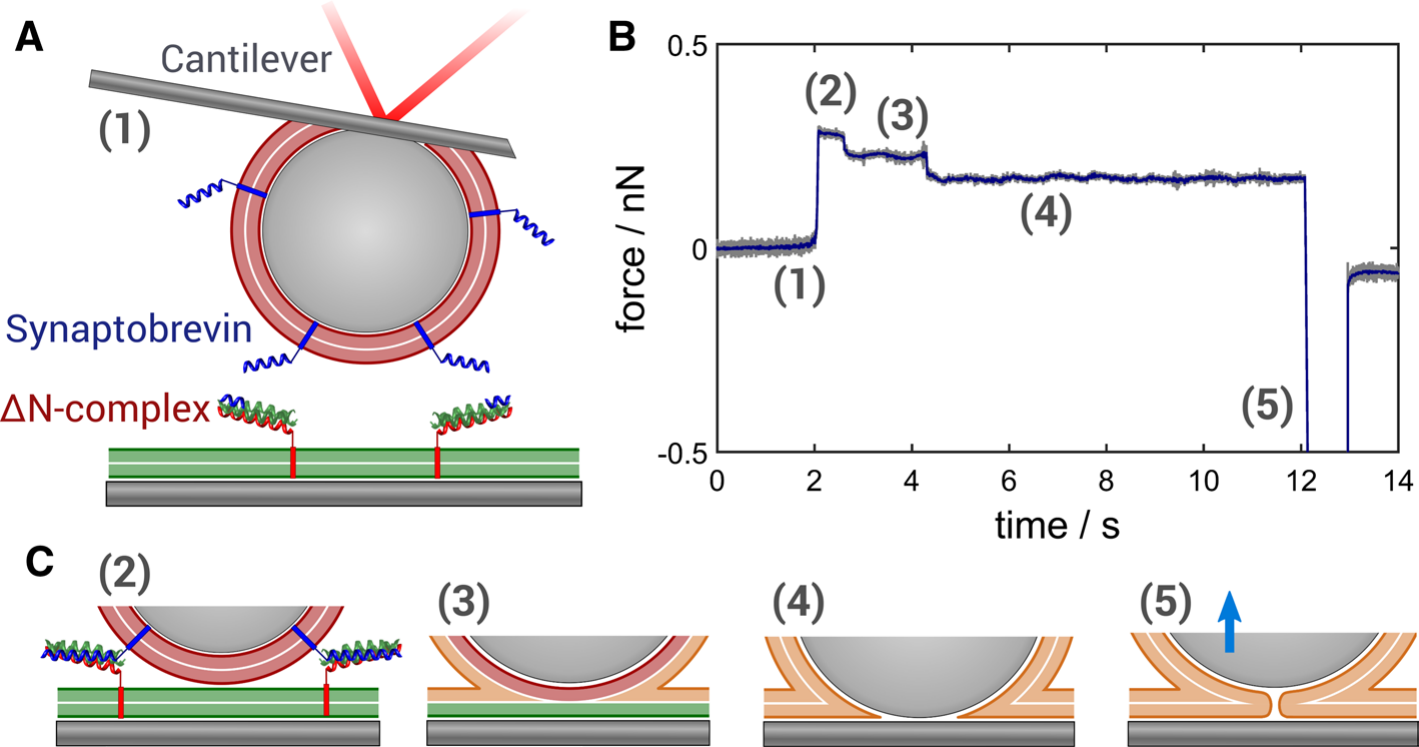
**a** Illustration of the colloidal probe microscopy setup. The colloidal probe is coated with a lipid bilayer and brought into contact with the opposing planar supported bilayer, both equipped with native fusion proteins as indicated. **b** Typical time trace of the fusion experiment. The steps can be assigned to specific transitions to fusion intermediates. **c** The following fusion events are discernible: (1) initial state, (2) SNARE complex formation (docking), (3) hemifusion, (4) full fusion, and (5) detachment from the surface (adapted from Oelkers et al. (2016) and Witt and Janshoff (2018))

A different approach is represented by colloidal probe microscopy (CPM), where the conventional tip of an AFM cantilever is exchanged by a colloidal particle to measure adhesion forces between two surfaces down to molecular resolution and also allows to exert compression forces. In general, CPM is ideally suited to examine the interaction between two microscopic surfaces with unprecedented resolution and dynamic range (Ducker et al. 1991; Kappl and Butt 2002; Lorenz et al. 2010; Pähler et al. 2013; Braunger et al. 2014; Witt et al. 2016; Lorenz et al. 2012; Krieg et al. 2018). The biggest advantage of colloidal probes over the commonly used sharp AFM tips is the reliable functionalization with biomolecules and even membranes. While colloidal probes permit visual inspection, successful functionalization of sharp tips cannot be directly confirmed. Colloidal probes were first used by Abdulreda et al. (2009) and later refined by Lorenz et al. (2010) as well as Oelkers et al. (2016) to measure forces involved in membrane merging in the presence of fusogens. Particularly, the force clamp technique improved our understanding of membrane fusion (Oelkers et al. 2016). Briefly, two opposing bilayers equipped with synaptobrevin on one side and the $\Delta$N-acceptor complex [composed of syntaxin 1, SNAP25, and a C-terminal fragment of synaptobrevin (Pobbati et al. 2006)] on the other side were prepared on a silica bead glued to a tipless cantilever (red) and on a planar glass substrate (green), respectively (Fig. 11a). The force clamp experiment comprises the following steps (Fig. 11b/c): the two opposing bilayers are rapidly approached until elastic contact is established. A defined load is applied and force feedback switched off allowing to monitor cantilever deflection as a function of time. After a defined contact time, the surfaces are separated by moving the cantilever rapidly away from the substrate. Frequent observations in the presence of SNAREs were stochastic steps of several nanometers towards the surface (Fig. 11b).

**Fig. 12:**
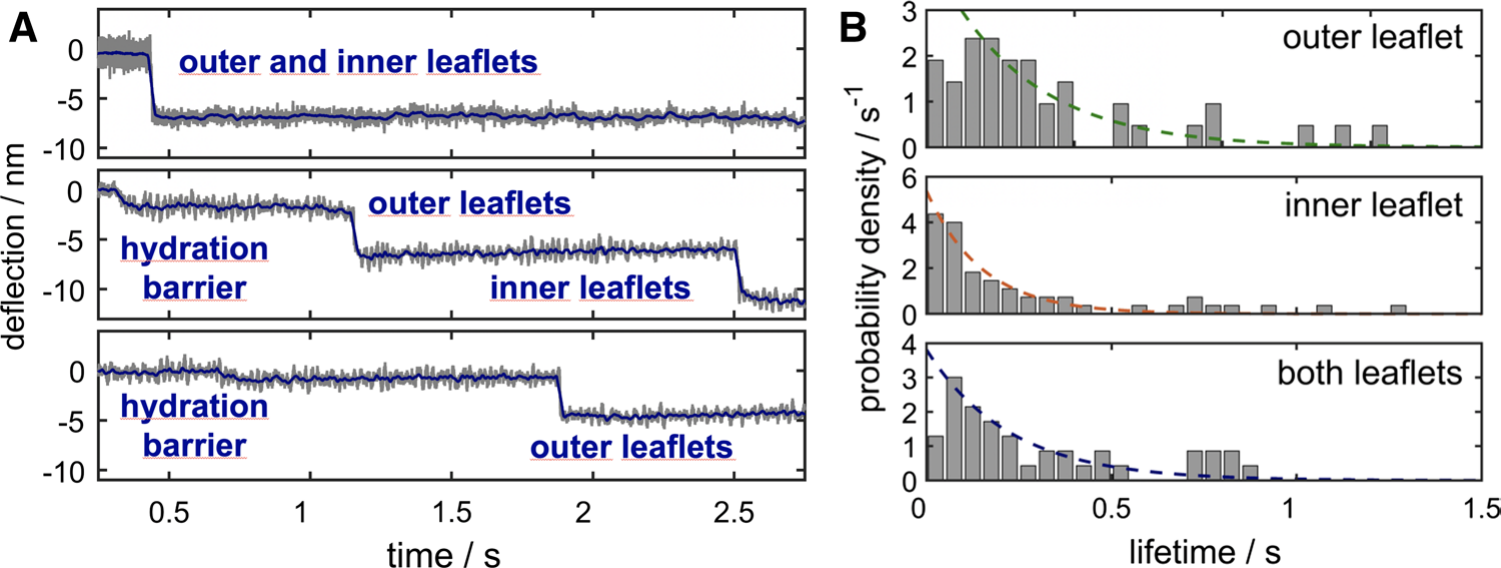
**a** Time traces of cantilever deflection during elastic contact of two membrane-coated surfaces. Three distinct scenarios are displayed: full fusion of the two lipid bilayers in a single step (top), consecutive fusion of first the outer and later the inner leaflets (center), and arrested hemifusion during the observed period of time (bottom). Frequently, small steps of 1–2 nm were found prior to fusion (center and bottom). These were assigned to the existence of a hydration barrier. **b** Lifetime histograms corresponding to the three cases. The dashed lines are monoexponential fits (adapted from Oelkers et al. (2016) and Witt and Janshoff (2018))

It was possible to unequivocally assign these discrete jumps or steps in the time traces of cantilever deflection to transitions between fusion intermediates (Oelkers et al. 2016). Among them, the clearest assignments were fusion events of only two leaflets (hemifusion or subsequent full fusion) and fusion of outer and inner lipid monolayers (one-step full fusion), characterized by jump distances corresponding to the thickness of a single membrane or two membranes, respectively. Smaller steps or instabilities below 2 nm were attributed to removal of the thin water layer between the two surfaces, i.e., crossing of the hydration barrier. Figure 12 shows representative time traces in which the various steps in membrane fusion were assigned. Full fusion occurred either as a one-step reaction (Fig. 12, top) or within two consecutive jumps (Fig. 12, center). Some deflection-time traces kept arrested either in a docked (not shown) or hemifused state (Fig. 12, bottom) during the contact time. In case of both leaflets fusing (full fusion), the overall step height was usually larger than 4–5 nm. Concomitantly, adhesion forces of up to 8 nN were found upon retraction of the cantilever. These large attractive forces could be attributed to van der Waals attraction between the two silica surfaces (vide supra).

**Fig. 13:**
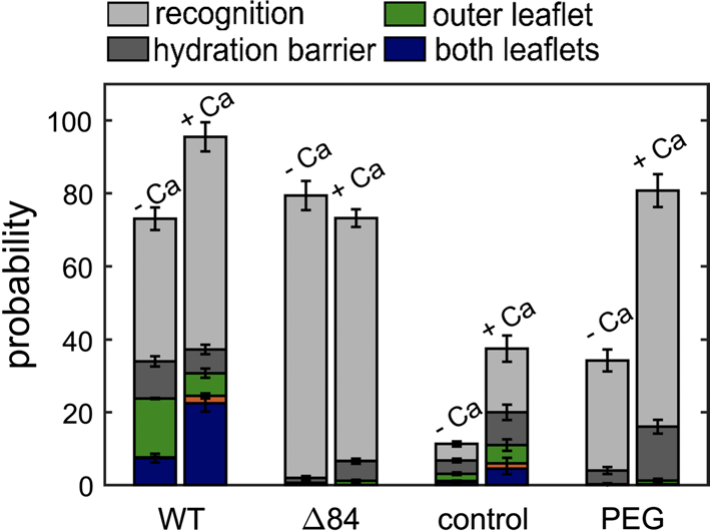
Occurrence of the indicated intermediate after a contact time of 10 s. Recognition refers to traces in which fusion was not observed, but significant attractive forces (> 50 pN) were recorded (adapted from Oelkers et al. (2016) and Witt and Janshoff (2018))

The probability that a given state (docked, hemifusion, and full fusion) was found after a contact time of 10 s is shown for a number of different conditions in Fig. 13. It was observed that bilayer merging of outer and inner leaflets was most likely with wild-type (WT) SNAREs in the presence of calcium ions. Interestingly, without calcium ions, membrane fusion was stalled after hemifusion and did not progress to full fusion. Recognition of opposing surfaces (identified by adhesion forces larger than 50 pN) required zippering of SNAREs and was largely independent of calcium ions. Interestingly, this was also observed for the $\Delta$ 84 syb mutant which only leads to incomplete formation of coiled-coil superhelices. However, neither hemifusion nor full fusion occurred if full zippering was prohibited by mutation in the sequence. Neat bilayers in the presence of calcium showed very few fusion-related events, which were mainly observed if defects in the supported bilayer existed. Notably, the addition of polyethylene glycol (PEG) increased the probability for membranes to overcome the hydration barrier, since it is known to reduce the hydration of interfaces.

**Fig. 14:**
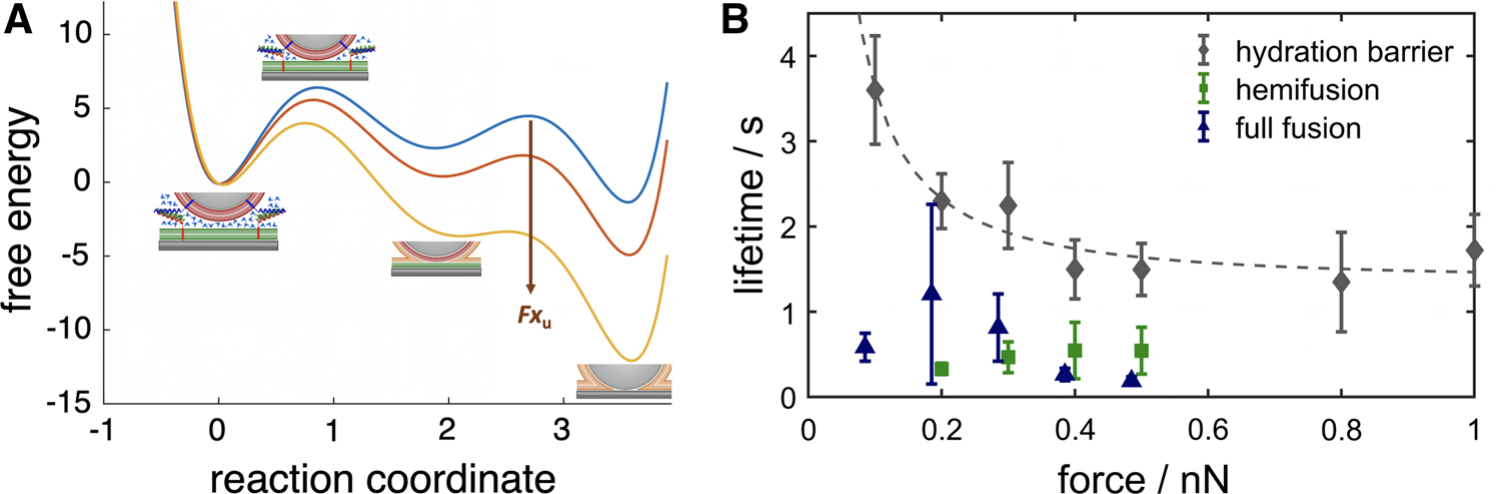
**a** Envisioned schematic illustration of how the energy landscape of membrane fusion might be affected by compressional forces. **b** Mean time from initial contact of the two membranes until crossing of the hydration barrier (gray), hemifusion (green), or full fusion (blue) as a function of applied force. The dashed line corresponds to a fit of Eq. 5 (adapted from Oelkers et al. (2016) and Witt and Janshoff (2018))

One of the benefits of colloidal probe microscopy is the possibility to apply an external load. The applied force reshapes the energy landscape and changes reaction rates over a large range (see Fig. 14a). While a constant loading force was applied, the corresponding average lifetime of the corresponding intermediate was measured, i.e., the time spans between initial elastic contact and pushing away the hydration layer, between removal of the hydration layer and hemifusion or one-step full fusion, and between hemifusion and consecutive full fusion, respectively. Figure 14b shows how the lifetime prior to removal of the hydration layer and between crossing of the hydration barrier, hemi-fusion and full fusion depend on the externally applied force. Essentially, dehydration of lipid bilayers was the only step of membrane fusion that showed a strong dependency on external load and turned out to be by far the slowest and hence rate-limiting step in SNARE-assisted membrane fusion (Oelkers et al. 2016).

The easiest way to describe the reaction rate under external load has been devised by Bell (Bell 1978) and first applied by Butt and Franz (2002) to describe the rupture of thin films attached to a surface. Adopting Bell’s theory for the geometry of CPM, the lifetime $\tau$ of a given intermediate depends on the external, compressive force *F*:

5 $$\begin{aligned} \tau (F)=\frac{1}{k_{\mathrm {L}} N_{\mathrm {L}}} \exp \left\{ -\frac{x^{\ddagger } A^{\ddagger } F}{k_{\mathrm {B}} T A}\right\} +\tau _\mathrm {os} \end{aligned}$$

with the rate at zero force:

6 $$\begin{aligned} k_{\mathrm {L}}=v \exp \left\{ -\frac{\Delta G^{\ddagger }}{k_{\mathrm {B}} T}\right\} . \end{aligned}$$

$\Delta G^{\ddagger }$ represent the energy barrier under consideration and $\tau _\mathrm {os}$ adds a force-independent time constant (offset) comprising, for instance, the lateral movement of proteins. $A^{\ddagger }$ is the size of the transition zone and $x^{\ddagger }$ is the distance to the energy barrier. $N_\mathrm {L}$ denotes the number of lipids in the contact zone and $A=\pi \left( 3 F R /\left( 4 E^{*}\right) \right) ^{2 / 3}$ the contact area assuming Hertzian contact. $E^{*}$ is the reduced Young’s modulus of the two bilayers. Since only the hydration barrier could be influenced significantly by the external force, it was hypothesized that the reaction coordinates of the consecutive steps such as fusion pore expansion might have a dominant in-plane component, orthogonal to the direction of applied force.

In conclusion, it was found that in the presence of SNAREs, the main energy barrier towards fused lipid bilayers originates from the hydration barrier between the opposing bilayers. After crossing of this barrier, full fusion can occur either directly or, with smaller probability, indirectly through a hemifusion intermediate state to generate a fully merged lipid bilayer.

## Conclusion

Taken together, colloidal probes in combination with optical microscopy, optical tweezers, or atomic force microscopy provide a versatile means to study membrane fusion with a minimum of labels/dyes. Additional information is gathered from applying external forces to explore the energy landscape of membrane merging. Prominent intermediate states could be unequivocally assigned and statistically quantified.

Moving forward, colloidal probes have a lot of potential to provide further insights into the details of membrane fusion, especially in the light of some recent innovations. Son et al. (2020) have demonstrated that circular averaging around a membrane-coated microsphere allows localization of fluorophores with sub-nanometer precision. Another very promising approach is to interface colloidal probes with living cells to form hybrid systems. The feasibility of this technique has been demonstrated for the study of macrophage phagocytosis (Bakalar et al. 2018; Joffe et al. 2020). There are also new methods emerging to manipulate colloidal probes. One promising example is acoustic force spectroscopy, where a standing acoustic wave is used to apply a force to microspheres (Sitters et al. 2015). The power of this method lies in the possibility to manipulate hundreds of probes at the same time allowing for high-throughput studies. Finally, it will be of high interest to reconstitute other parts of the fusion machinery alongside the core SNARE complex to address fundamental questions like why exocytosis of synaptic vesicles is so much faster compared to other fusion processes, for instance in endocrine cells (Kreutzberger et al. 2019).
